# Neurological deterioration after posterior decompression of an unrecognised calcified thoracic disc: recovery following endoscopic discectomy

**DOI:** 10.1093/jscr/rjag970

**Published:** 2026-10-27

**Authors:** Ralph J Mobbs, Edwin H Y Lui, Joseph Reidy

**Affiliations:** Faculty of Medicine and Health, University of New South Wales, High St, Kensington, NSW 2052, Australia; NeuroSpine Clinic, Prince of Wales Private Hospital, Barker St, Randwick, NSW 2031, Australia; NeuroSpine Surgery Research Group (NSURG), Barker St, Randwick, NSW 2031, Australia; Department of Neurosurgery, Prince of Wales Private Hospital, Barker St, Randwick, NSW 2031, Australia; Faculty of Medicine and Health, University of New South Wales, High St, Kensington, NSW 2052, Australia; NeuroSpine Clinic, Prince of Wales Private Hospital, Barker St, Randwick, NSW 2031, Australia; NeuroSpine Surgery Research Group (NSURG), Barker St, Randwick, NSW 2031, Australia; Department of Neurosurgery, Prince of Wales Private Hospital, Barker St, Randwick, NSW 2031, Australia; NeuroSpine Surgery Research Group (NSURG), Barker St, Randwick, NSW 2031, Australia; Department of Neurosurgery, Prince of Wales Hospital, Barker St, Randwick, NSW 2031, Australia

**Keywords:** calcified thoracic disc, thoracic myelopathy, endoscopic discectomy, laminectomy, neurological deterioration

## Abstract

Calcified thoracic disc herniation is uncommon but carries substantial operative risk. A 67-year-old man with 12 months of progressive myelopathy from a T7-T8 disc herniation underwent posterior laminectomy and instrumented fusion without preoperative computed tomography (CT). He developed near-complete paraplegia, with lower-limb power of Medical Research Council grade 0–1/5. Subsequent CT demonstrated a densely calcified ventral disc. Following transfer, full-endoscopic transforaminal discectomy was performed to address the ventral compression without spinal cord retraction. By three months after the revision procedure, power improved to grade 4–4+/5 and he regained ambulation using a walking frame. Thoracic disc herniations being considered for surgery should be characterised with CT as well as magnetic resonance imaging. Calcified lesions warrant subspecialty review and an approach that directly addresses the ventral pathology. In selected patients, endoscopic discectomy may achieve effective decompression with limited access morbidity.

## Introduction

Symptomatic thoracic disc herniation is rare, and approximately 40% of surgically relevant lesions are calcified [1]. When the lesion is central, progressive myelopathy may dominate the clinical picture. The narrow thoracic canal and vulnerable segmental blood supply leave little tolerance for cord manipulation during decompression [1–3].

Magnetic resonance imaging (MRI) defines the level and severity of cord compression, intramedullary signal change and associated soft-tissue pathology. Computed tomography (CT), however, is essential for determining whether a disc is soft, calcified or ossified and for defining the surrounding osseous anatomy. This distinction materially changes operative planning, since a rigid, dura-adherent fragment cannot be safely mobilized through a corridor requiring cord retraction [1, 4]. Posterior laminectomy alone carries an unacceptable risk of neurological deterioration, since dorsal decompression does not address a ventral lesion [2, 3]. Central calcified lesions therefore generally require an anterior, anterolateral or lateral route, although a carefully selected posterolateral endoscopic corridor can also achieve ventral decompression without cord retraction [4–10].

We report severe postoperative paraparesis after posterior decompression and fusion for an unrecognised calcified T7-T8 disc, followed by meaningful neurological recovery after endoscopic discectomy. The case highlights the need for preoperative CT, subspecialty referral for calcified lesions, and a surgical corridor directed to the ventral compression.

## Case report

A 67-year-old man presented to another institution with a 12-month history of progressive thoracic myelopathy, characterized by gait and balance disturbance with brisk lower-limb reflexes (3+/4), preserved power (5/5), and no sensory or sphincter disturbance. MRI demonstrated a large T7-T8 disc herniation with marked ventral compression (Fig. 1). Preoperative CT was not obtained, so the disc’s calcification was not defined before surgery.

**Figure 1:**
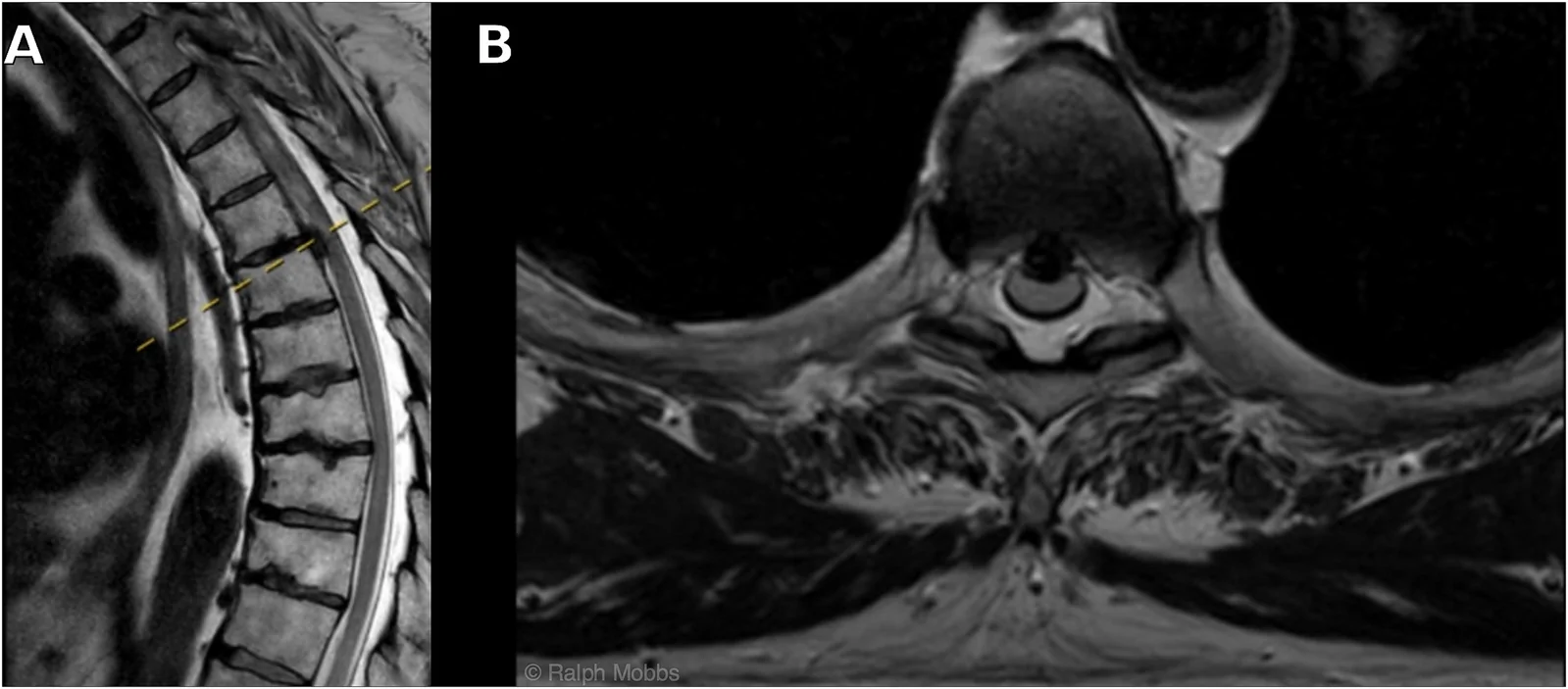
Preoperative MRI prior to the initial surgery. (A) Mid-sagittal T2-weighted MRI. (B) Axial T2-weighted MRI.

He underwent posterior T7-T8 laminectomy and instrumented fusion at the referring institution. Immediately after surgery he developed acute paraparesis, bilateral lower-limb power 0–1/5 (flicker contraction only, without antigravity movement), a T7 sensory level, and bladder dysfunction requiring an indwelling catheter; he remained functionally paraplegic with no improvement over the following 6–8 weeks, when he was transferred to our spinal injuries unit. Examination classified the injury as American Spinal Injury Association (ASIA) Impairment Scale C (motor score 60/100: 50/50 upper limbs, 10/50 lower limbs), with sacral sensory sparing and retained partial bladder sensation. CT confirmed a densely calcified T7-T8 disc remaining ventral to the cord (Fig. 2). MRI before revision surgery demonstrated persistent anterior cord compression despite the laminectomy and fusion (Fig. 3A).

**Figure 2:**
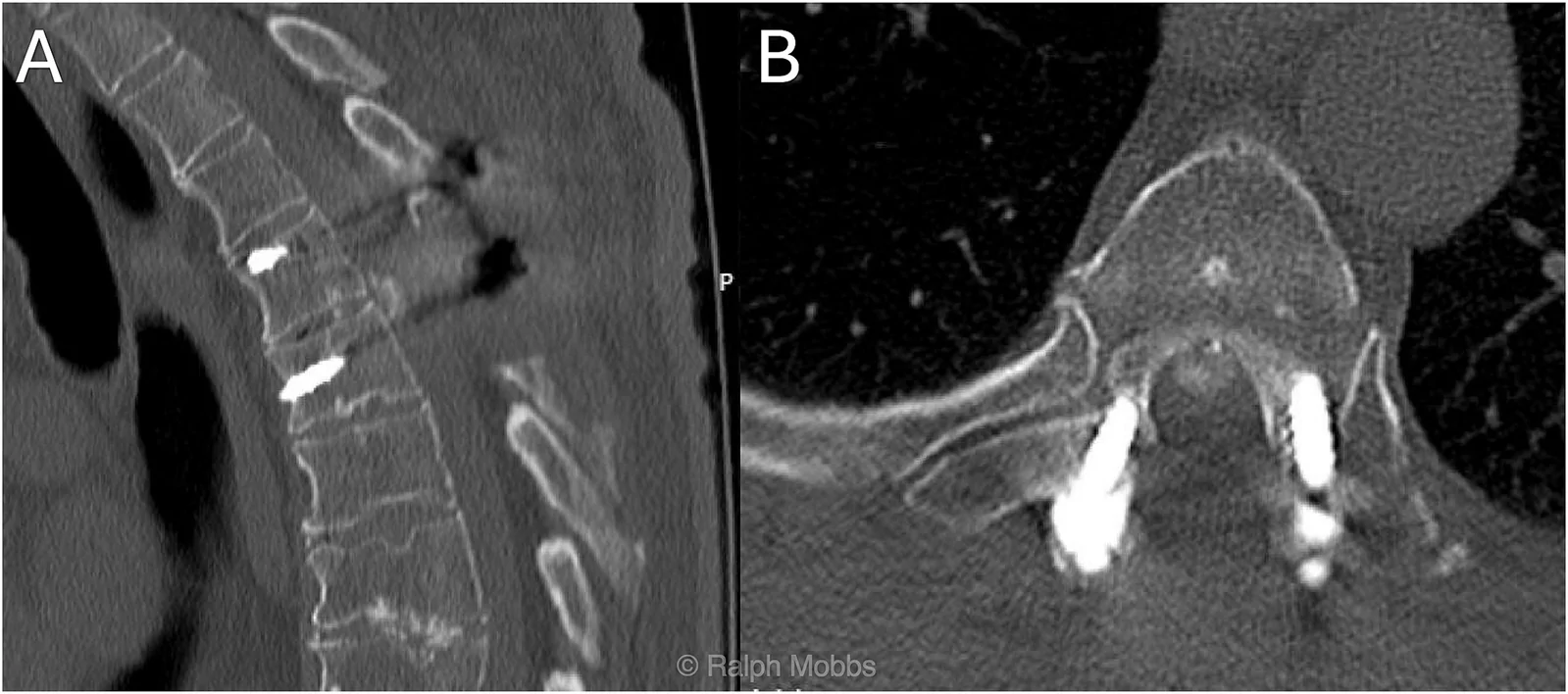
CT obtained after the index operation and before revision surgery, demonstrating calcified T7-T8 disc. (A) Sagittal CT demonstrating the calcified fragment in relation to the adjacent vertebral bodies and posterior instrumentation. (B) Axial CT.

**Figure 3:**
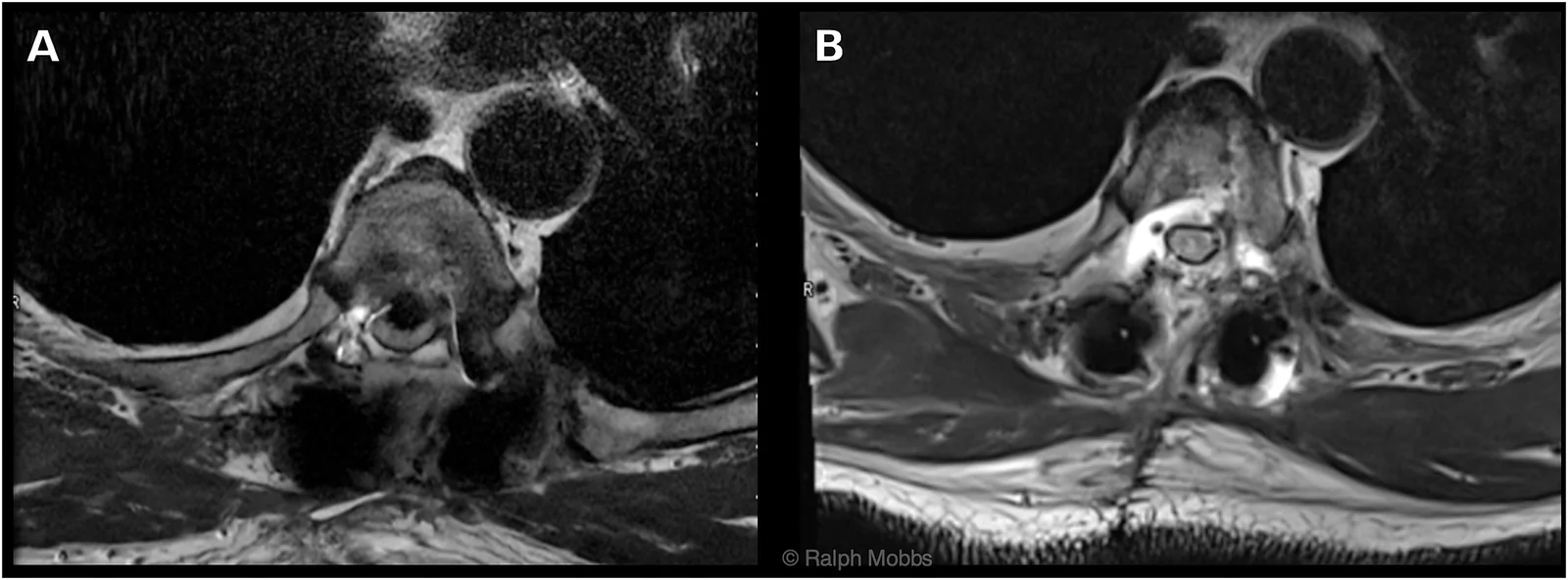
Axial T2-weighted MRI before and after full-endoscopic discectomy. (A) Preoperative MRI prior to endoscopic discectomy, demonstrating hardware from the posterior fusion and persistent anterior spinal cord compression. (B) Postoperative MRI following endoscopic discectomy, demonstrating the right-sided surgical tract and complete anterior decompression of the spinal cord.

Following subspecialty review, revision surgery was undertaken approximately two months after the index operation. With the patient prone under general anaesthesia, a full-endoscopic right-sided transforaminal approach to T7-T8 using a 6.9 mm × 180 mm endoscope (Elliquence, Matrix Medical) was performed, per a previously described technique [12]. Prior instrumentation made fluoroscopic identification of the docking point and trajectory markedly more difficult than in non-instrumented spine; anteroposterior and lateral fluoroscopy confirmed the foraminal trajectory and instrument position throughout (Fig. 4A–C). Foraminoplasty was performed with a 3 mm high-speed drill, and the calcified fragment, found to be adherent to the dura, was completely removed using drill, pituitary rongeur and radiofrequency probe, without cord retraction; intraoperative neuromonitoring was not used.

**Figure 4:**
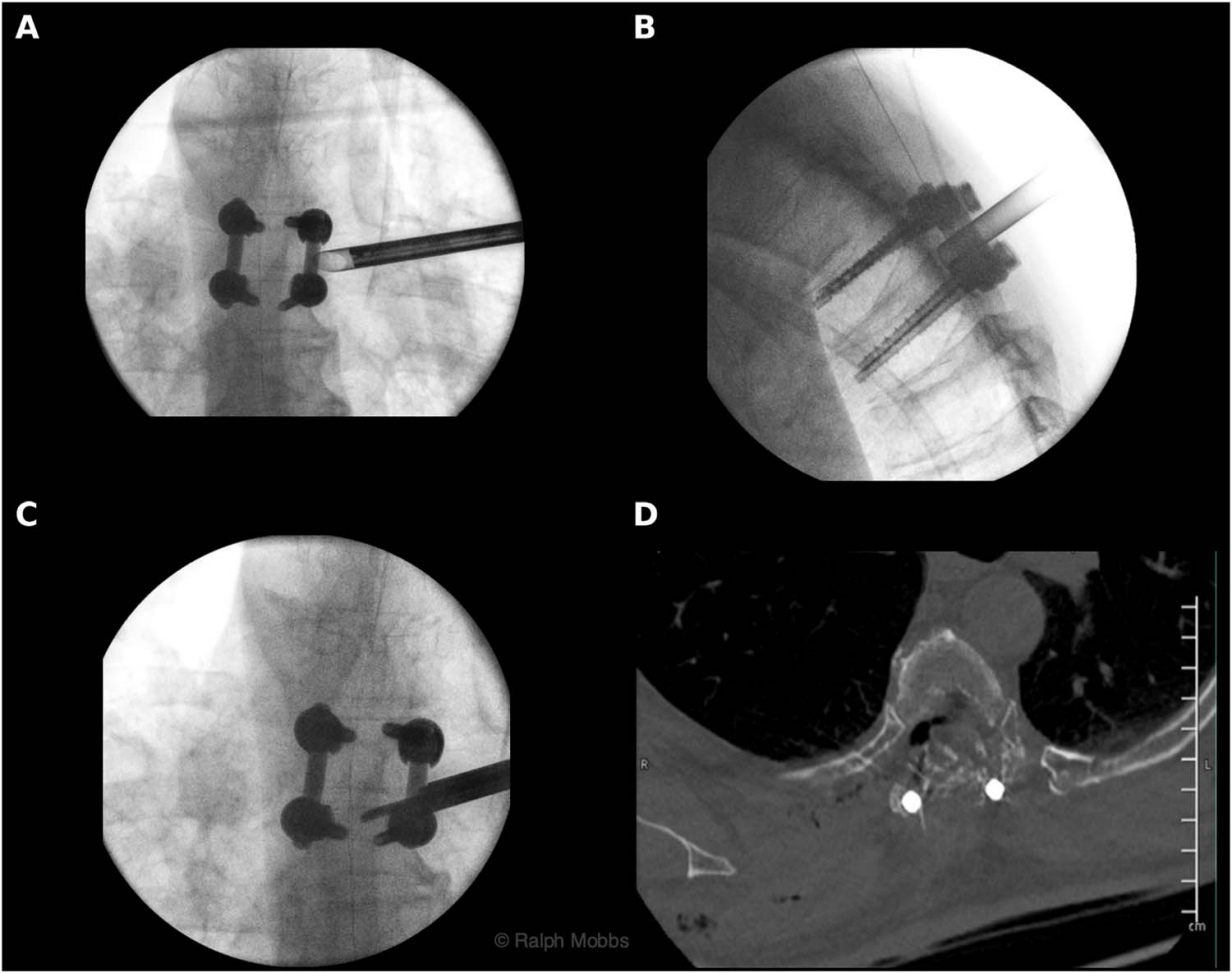
Intraoperative fluoroscopy and postoperative CT. (A) Anteroposterior fluoroscopic image showing the working cannula positioned in the T7–T8 foramen. (B) Lateral fluoroscopic image confirming the working trajectory. (C) Anteroposterior fluoroscopic image showing a pituitary rongeur performing the discectomy. (D) Postoperative axial CT demonstrating the right-sided approach angle from a skin entry point approximately 8 cm lateral to the midline.

Operative time was 130 minutes, with minimal (<1 mL) blood loss and no intraoperative complications. Postoperative CT confirmed the right-sided approach (Fig. 4D), and postoperative MRI confirmed complete anterior decompression and fragment removal (Fig. 3B).

Neurological recovery was gradual. Lower-limb power improved from 0–1/5 immediately after the index operation to 4–4+/5 at three months after the revision procedure, with a persistent T7 sensory level and a spastic gait pattern. At the latest assessment, he was ambulant using a walking frame.

## Discussion

This case demonstrates the consequence of an unrecognized calcified fragment: the index laminectomy created dorsal space but left the ventral lesion untouched, and in the literature, subsequent cord shift, manipulation or vascular compromise may have been implicated in neurological deterioration after such procedures [1, 3–5]. We advocate thin-slice CT alongside MRI for all surgical thoracic disc herniations. Failure to identify calcification may lead to a corridor with inadequate ventral access.

Full-endoscopic approaches to the thoracic spine continue to expand [11–13], achieving direct ventral decompression through a posterolateral corridor, with reduced tissue disruption and, in selected cases, no need for fusion [6–10]. This approach is not universally applicable: central giant calcification, intradural extension, severe dural adherence or unfavorable anatomy may still favor an open approach. Furthermore, the steep learning curve demands surgeons highly experienced in both thoracic and endoscopic techniques.

Several factors could have contributed to the acute deficit, intraoperative cord manipulation, vascular injury, epidural haematoma, hypotension, direct trauma and cord oedema among them, and the absence of neuromonitoring records from the index procedure means the precise mechanism cannot be confirmed. Persistent ventral compression is, however, the most likely explanation. As a single case, this does not establish which mechanism predominates, the recovery observed most likely reflects decompression together with time and rehabilitation.

## Conclusion

All patients being considered for surgery for a thoracic disc herniation should undergo CT in addition to MRI. CT identifies calcification and defines the osseous anatomy that determines whether a proposed corridor can safely reach the ventral pathology. When a central calcified lesion is identified, subspecialty review is advisable before surgery.

An isolated posterior laminectomy should not be used to treat a central calcified ventral thoracic disc, since it does not remove the causative lesion; the operation should instead provide direct anterior or lateral access, or a carefully planned posterolateral corridor that avoids spinal cord retraction. In this selected case, full-endoscopic discectomy achieved effective anterior decompression through a limited working channel without further fusion, and the patient regained meaningful function after a severe deficit. This was performed by a surgeon experienced in endoscopic thoracic spine surgery; the approach is not universally applicable, and a single case cannot disentangle the relative contributions of decompression, rehabilitation, and spontaneous recovery, or establish that timely revision will reliably reproduce this outcome in others. Nonetheless, this case supports endoscopic decompression as a viable option in carefully selected patients, and further experience may help define which patients are best suited to this approach.
